# Pediatric empiric antibiotic use and resistance patterns at a single tertiary care hospital in Tanzania

**DOI:** 10.1186/s12887-026-07261-z

**Published:** 2026-07-08

**Authors:** Anneka M. Hooft, Kum Su Sunny Hwang, Raya Mussa, Prachi Singh, Hendry Robert Sawe, Juma A. Mfinanga, Newton Addo, Teresa Kortz, Joel Manyahi

**Affiliations:** 1 Departments of Emergency Medicine and Paediatrics, University of California, San Francisco, San Francisco, CA USA; 2 Institute for Global Health Sciences, University of California, San Francisco, San Francisco, CA USA; 3 Department of Paediatrics, University of California, San Francisco, San Francisco, CA USA; 4 Department of Emergency Medicine, Muhimbili University of Health and Allied Sciences, Dar es Salaam, Tanzania; 5 Department of Emergency Medicine, Muhimbili National Hospital, Dar es Salaam, Tanzania; 6 Department of Emergency Medicine, University of California, San Francisco, San Francisco, CA USA; 7 Department of Microbiology and Immunology, Muhimbili University of Health and Allied Sciences, Dar es Salaam, Tanzania

**Keywords:** Antibiotic resistance, Pediatrics, Sub-Saharan Africa, Antimicrobial stewardship, Antibiogram

## Abstract

**Background:**

Despite growing microbiology capacity in Tanzanian public hospitals, pediatric-specific antibiograms remain rare, and patterns of empiric antibiotic use in emergency care are poorly described compared to high-income settings. Understanding resistance profiles and empiric prescribing practices is essential for developing successful pediatric antimicrobial stewardship (AMS) programs.

**Objectives:**

To assess baseline pediatric empiric antibiotic use in the emergency medical department (EMD) and microbiology results from children at Muhimbili National Hospital (MNH), a tertiary care center and referral hospital in Dar es Salaam, Tanzania.

**Design:**

We retrospectively reviewed the electronic medical records of children seen in the EMD over a one-year period to document empiric antibiotic use and indications. Blood and urine cultures were analyzed to construct a pediatric-specific antibiogram and assess resistance patterns.

**Results:**

Of 2,316 patients prescribed antibiotics, lower respiratory infection (*n* = 532, 23%), prophylaxis (*n* = 380, 16%), and upper respiratory infection (*n* = 277, 12%) were the most common single indications for use. Ceftriaxone was the most frequently used antibiotic (*n* = 1,658, 72%). Microbiology data revealed low susceptibility of gram-negative isolates to ceftriaxone: 20% (10/49) in *K. pneumoniae* and 18% (9/51) in *E. coli*. Over 90% of Enterobacterales met criteria for multidrug resistance (MDR).

**Conclusions:**

Ceftriaxone was the most common empiric antibiotic, particularly for lower respiratory infections. Antibiotic treatment selection aligned with local sepsis guidelines, emphasizing broad criteria for therapy rather than national, source-directed infectious disease treatment guidelines. Resistance data demonstrated frequent ceftriaxone resistance among gram-negative organisms. Future studies are needed to understand clinicians’ use of bacterial cultures and clinical outcomes in children with resistant isolates. Quality improvement initiatives should incorporate regular review of antibiotic prescribing patterns and microbiology data to ensure treatment guidelines reflect local epidemiology.

**Supplementary Information:**

The online version contains supplementary material available at https://doi.org/10.1186/s12887-026-07261-z.

## Introduction

Antibiotic use in low- and middle-income countries (LMICs) is steadily increasing, with concern for rising rates of antimicrobial resistance (AMR) [1–3]. Sub-Saharan Africa (SSA) bears both the highest burden of deaths attributable or associated with AMR [4]. This region of the world also has the highest rates of morbidity and mortality from sepsis, with over two million deaths per year, which is likely an underestimate [5]. Despite the disproportionately large burden of infectious diseases and antimicrobial resistance, the frequency and characteristics of antibiotic use, bacterial disease burden, and AMR profiles in pediatric patients are not well tracked [6]. 

The use of empiric antibiotic therapy is common in children who present with fever in SSA, with the frequency of antibiotic prescription for these children as high as 95% in some settings [7–9]. In Tanzania, a point prevalence study demonstrated that children under two years of age are more likely to receive antibiotic treatment [10], while others have found high rates of guideline-discordant use of ceftriaxone [11]. Differentiating the etiology of fever in children can be especially challenging due to overlap in the clinical presentations of common viral, bacterial, and parasitic infections, limited laboratory testing capacity, and a lack of localized epidemiologic data [12]. Clinicians may not feel comfortable discharging children without antibiotic treatment due to the inability to ensure adequate follow-up, health system limitations, and a desire to prevent poor outcomes [13, 14]. Patterns of antibiotic prescription in referral-level hospitals are less well described. They may differ from those in the primary care setting, given the availability of more specialized clinical providers, the increased likelihood of pretreatment, and higher acuity.

Prior studies in academic microbiology labs in Tanzania have demonstrated increasing AMR among pediatric patients, including extended-spectrum beta-lactamase (ESBL)-producing and multidrug-resistant organisms [15–21]. Proportions of these resistant organisms are likely underestimated, given the decreasing cost of and increasing access to broad-spectrum antibiotics, while clinical microbiology laboratory capacity remains limited [15–21]. Despite these studies in more controlled, academic settings, the capacity for microbiologic culture and susceptibility testing is not widely available in public hospitals. Additionally, results are often not provided to the clinical team in a timely manner due to a lack of data systems infrastructure, leading to infrequent use of microbiology data to tailor antibiotic selection [22]. Antibiotic treatment guidelines are often based on overlapping clinical signs and symptoms, given the paucity of studies on the etiology of fever, the true prevalence of bacterial illness, and bacterial resistance patterns in the public hospital setting in Tanzania and other LMICs (Table 1). All of these factors may contribute to high frequencies of empiric antibiotic use.

**Table 1:** Recommendations for antibiotic treatment of common pediatric infections from the Tanzanian Standard Treatment Guidelines, the Emergency Medical Association of Tanzania, and the Muhimbili Emergency Medical Department Sepsis Guidelines

Diagnosis		1st line	Alternatives/2nd line
Pneumonia	Non-severe	Amoxicillin	Cotrimoxazole
	Severe	Benzyl penicillin, then amoxicillin	Ampicillin + gentamicin
	Very severe	Ampicillin + gentamicin	Ceftriaxone
	Severe with aspiration	Add metronidazole	
	Atypical	Erythromycin	Azithromycin OR clarithromycin
Upper respiratory infection (URI)	Acute otitis	Phenoxymethylpenicillin	Amoxicillin-clavulanate OR azithromycin (if PCN allergy) OR clarithromycin OR ceftriaxone
	Sinusitis	Amoxicillin clavulanate	Azithromycin OR clarithromycin OR ceftriaxone
	Strep pharyngitis	Benzyl penicillin	Penicillin G
Urinary Tract Infection (UTI)	Uncomplicated	Nitrofurantoin	Amoxicillin-clavulanate
	Pyelonephritis	Ciprofloxacin	Ceftriaxone
	Urosepsis	Gentamicin + Ceftriaxone-sulbactam	Gentamicin + piperacillin-tazobactam OR meropenem
Gastrointestinal	Typhoid	Ceftriaxone	
	Enteritis	Based on specific pathogen	
	Appendicitis	Ceftriaxone + metronidazole	
Neurologic	Meningitis	Benzyl penicillin and chloramphenicol	Ceftriaxone OR cefotaxime
Skin infection	Cellulitis/Abscess	Erythromycin	Flucloxacillin
Sepsis		STG: Ampicillin + cloxacillin + gentamicin^†^ EMD: Ceftriaxone + Metronidazole^‡^	Gentamicin + ceftriaxone OR ceftriaxone-sulbactam OR piperacillin-tazobactam OR meropenem + vancomycin (if immunosuppressed)^θ^
Prophylaxis	Trauma	No recommended routine antibiotic treatment	

*PCN *penicillin, *MNH *Muhimbili National Hospital, *EMD *Emergency Medical Department, *STG *Standard Treatment Guidelines

^**†**^No pediatric-specific criteria. Based on 2 + Quick Sequential Organ Failure Assessment (qSOFA) criteria: hypotension, elevated respiratory rate, altered mental status +/- culture-proven bacterial infection

^‡^ Based on 2 + systemic inflammatory response (SIRS) criteria by age: elevated heart rate, elevated respiratory rate, fever, leukocytosis, hypotension + suspected or proven infection

^θ^Meropenem + vancomycin is the only recommended 2nd -line treatment in the MNH EMD guideline. Both this and the others listed as 2nd line are included in the Tanzania STG. STG recommends escalation of therapy if no clinical improvement after ≥ 24 h

The World Health Organization (WHO) has recently emphasized the need for antimicrobial stewardship (AMS) programs in LMICs and has developed specific guidelines for creating and maintaining them [23]. In concordance with these recommendations, the Tanzanian Ministry of Health has recently established a nationwide AMS program [24]; however, this has been difficult to fully implement in practice. Data from Tanzanian public health facilities on antibiotic use and AMR have neither been routinely collected nor analyzed. Through a capacity-building grant from the Fleming Fund since 2020, MNH has the materials, personnel, and training to perform and analyze bacterial culture and resistance profiles for blood and urine samples in its microbiology laboratory. Thus far, these data have neither been fully analyzed nor synthesized to create an antibiogram. Results also do not routinely inform clinical decision-making due to limitations in medical record-keeping and clinician notification systems.

This study aimed to review current antibiotic use guidelines, to describe empiric antibiotic use data, and to analyze available microbiology results for antimicrobial resistance patterns in pediatric patients evaluated at MNH. These findings will inform the reestablishment of a pediatric AMS committee and aid in the review and improvement of clinical guidelines, the development of educational initiatives, and the creation of additional targeted interventions as part of a larger quality improvement initiative.

## Methods

This was a retrospective medical records review study in partnership with MNH, Muhimbili University of Health and Allied Sciences (MUHAS), and the University of California, San Francisco, as part of an ongoing quality improvement initiative that started in March 2022 to reestablish a pediatric antimicrobial stewardship program at MNH in Dar es Salaam, Tanzania.

### Study setting

Muhimbili National Hospital is a government-funded, public hospital in Dar es Salaam, Tanzania, serving as a national referral hospital and a primary teaching hospital for MUHAS. The Emergency Medicine Department (EMD) at MNH is a 24-hour, full-capacity facility that offers basic laboratory testing, plain-film radiographs, bedside ultrasound, and cardiorespiratory monitoring. Approximately 60,000 patients visit the EMD each year, and about one-quarter are children aged 28 days to 15 years; 75% of those seen are admitted to the hospital for further management. Clinicians in the EMD primarily use a local sepsis treatment guideline, the Tanzania Standard Treatment Guidelines (STG) [25], and the Emergency Medical Association of Tanzania mobile app (also based on the STG) [26] to guide antibiotic prescribing. These guidelines for common pediatric infectious conditions are summarized in Table 1. Since 2020, the MNH Central Processing Laboratory (CPL) has had the capacity to culture blood, urine, and wound samples using equipment and training resources provided through a grant from the Fleming Fund.

### Antibiotic use in the EMD

A study research assistant conducted a daily retrospective review of all children seen in the EMD the previous day, using the electronic medical records (EMR) and linked electronic prescribing systems to analyze prescriptions.

#### Inclusion criteria

We reviewed all visits for children aged 28 days to 15 years (children under 28 days are seen in the neonatal unit) from May 19, 2022, through April 30, 2023, who were prescribed at least one antibiotic in the EMD.

#### Exclusion criteria

Children seen for indications who did not receive antibiotics.

#### Data collection

Data collected from the EMR were extracted and recorded in a REDCap data entry form by a study research assistant. The data collection form was modeled after the WHO Antimicrobial Stewardship Review Form [23]. This form included information taken from the provider note, including demographics (age, gender, weight), antibiotic history (treatment prior to EMD visit, current antibiotic use), referral status, antibiotic agents administered in the EMD and/or prescribed upon discharge, indication(s) for antibiotic use, and the patient’s disposition. Indication for antibiotic use was abstracted from the treating clinician’s assessment and entered by the research assistant as a categorical variable for type of infection. We additionally reviewed and recorded the antibiotic, dose, and route from the prescription ordering system. Data quality was reviewed by the study manager and principal investigators intermittently via direct observation, verbal confirmation with the treating clinician, and correlation with the patient’s clinical notes. All treatment indications entered as “Other” were also reviewed by the study principal investigators and converted to an existing indication category where appropriate.

### Culture processing, identification, and resistance profiling

Our laboratory technician and project data managers retrospectively collected all bacterial culture and sensitivity results recorded by the MNH CPL for all pediatric patients undergoing microbial analysis.

#### Inclusion criteria

All pediatric patients (ages 28 days to 15 years) at MNH with culture samples collected and processed from March 1, 2022, to February 28, 2023.

#### Exclusion criteria

We excluded patients in the labor and delivery and neonatal wards, given these patients fall outside of the pediatric empiric treatment guidelines, are seen by neonatal providers, and have unique microbiologic exposures and profiles. While we initially reviewed all available data for any culture result. Given the high variability in sample source for wound cultures and other superficial isolates with questionable interpretation and clinical relevance, including a large number of samples obtained from surgical wounds, we narrowed our study sample to blood and urine cultures only.

#### Microbiology lab procedures

Clinicians collected all specimens, and microbiological procedures were performed at the MNH CPL in accordance with standard operating procedures. Urine samples were collected by both catheter and clean-catch methods, and the collection method was not routinely documented in the microbiology data system. Blood cultures were incubated on the BD BACTEC™ 200 blood culture system. Cultures identified with positive growth were cultured on sheep blood agar, chocolate agar, and MacConkey agar. Quantitative culture for urine was plated onto cystine lactose electrolyte-deficient agar using a 1 µL calibrated loop; pure growth colonies > 1 × 10^5 CFU/mL were considered significant bacteriuria. Sheep blood agar, MacConkey agar, and cystine lactose electrolyte-deficient agar were incubated at 37 °C for 20 h. Chocolate agar was incubated in a 10% atmosphere of CO₂, at 37 °C for 20 h. Isolates were identified by morphology, Gram stain, and conventional biochemical tests, including API 20E for Enterobacterales and Staphaurex™ for *S. aureus*. Antimicrobial susceptibility testing was performed using the Kirby-Bauer disk diffusion method, and interpretation followed the Clinical and Laboratory Standards Institute (CLSI) guidelines, 32nd Edition [27]. Isolates with known intrinsic resistance to certain agents were not tested against those agents. The choice of antibiotics used for susceptibility testing was based on MNH Standard Operating Procedures (see Supplementary File 1). Due to disk stockouts at times, not all samples were tested against all recommended agents.

#### Contaminates

For all mixed-growth urine cultures, patients were advised to repeat sample collection per the facility’s standard operating procedure. For this study, all urine cultures with mixed or no growth were categorized as negative. For blood cultures, we followed the lab’s standard operating procedure, which considered all coagulase-negative Staphylococcus, Bacillus, and Corynebacterium species contaminants because only one set of blood cultures was performed. We considered all contaminants as negative results for analysis.

#### Data collection

While the MNH CPL routinely uses WHONET for data entry, a project laboratory technician assisted with this process through the creation of a separate pediatric-specific WHONET database, to which we added and organized all culture and antimicrobial susceptibility testing results for laboratory-based surveillance of antimicrobial resistance in these patients [28]. Information entered included patient age, gender, specimen type, patient location in the hospital at the time of culture collection, culture results, bacterial isolates, and susceptibility results. Data cleaning and deduplication were performed in WHONET. To reduce bias and overestimation of resistance frequency in the antibiogram (Table 5), we followed CLSI standards to include only the first isolate result per patient over the course of the study period, regardless of source site [29]. To determine characteristics at the patient level (Tables 4 and 6), we included only the first positive isolate per patient, regardless of species, to avoid overrepresentation of participants with multiple positive isolates during the study period. In Table 4, for participants with multiple samples with no growth, we also defined characteristics at the time of first sample collection by date.

### Analysis

We performed descriptive analysis of both the EMD antibiotic use and microbiologic data from the MNH lab using the R v4.2 *tidyverse*, *gtsummary*, and *knitr* packages to summarize characteristics, frequencies, and proportions, including missing data [30]. 

For microbiologic data, we also analyzed blood and urine samples within WHONET to provide descriptive characteristics for the cohort and total frequencies for organisms isolated. In consultation with a pediatric infectious diseases specialist and clinical microbiologist (PS and JM), as well as members of the microbiology and clinical teams at MNH, a resistance report and antibiogram were developed following the Clinical and Laboratory Institute M39-A4 guideline [31], based on bacterial culture results and resistance profiles identified in samples from all pediatric blood and urine collected at MNH (Table 4). Because this guideline recommends a minimum of *N* = 30 for inclusion of isolated species in the antibiogram, we combined blood and urine results. We then grouped less frequently encountered species into larger categories (e.g., Other Enterobacterales, all *Acinetobacter* species) to increase total counts. Methicillin-resistant *Staphylococcus aureus* (MRSA) was classified based on a zone of inhibition of ≤ 21 mm to cefoxitin, given the lack of availability of oxacillin or methicillin in the CPL, with results reported as oxacillin resistance as per CLSI convention [27, 31]. Any intermediate results were grouped as resistant.

We defined multidrug resistance (MDR) according to the European Centre for Disease Prevention and Control and the Centers for Disease Control and Prevention criteria [31]. For Enterobacterales as a group, this was defined as resistance to ≥ 1 antibiotic agent in ≥ 3 classes of antibiotics for each respective group, excluding those antibiotic classes to which a species has known intrinsic resistance (e.g., ampicillin for all Enterobacterales other than *E. coli*). For MDR *P. aeruginosa*, this was similarly defined as non-susceptibility to *≥ 1* agent in ≥ 3 of the 5 antibiotic classes of antipseudomonal agents. Due to capacity constraints, induced clindamycin resistance was not assessed. S. *aureus* was defined as MDR if resistance was demonstrated to ≥ 3 classes of antibiotics with known anti-staph activity. For MDR analyses, samples lacking test results for a given agent were considered susceptible to that agent. Due to resource limitations, resistant and MDR organisms did not have further confirmatory or genetic testing to confirm mechanisms of resistance [24]. 

## Results

### Empiric antibiotic use

Among 7734 total pediatric patients evaluated in the EMD between May 2022 and April 2023, 2324 (30%) received at least one antibiotic. Of these, 45% (*n* = 1037) were younger than 2 years of age, 30% (*n* = 690) were between 2 and 5 years, and the remaining 25% (*n* = 589) were between 6 and 14 years, with 1.2% (*n* = 29) missing documentation of the number of antibiotics received (Table 2). The most used antibiotic was ceftriaxone (*n* = 1653, 72%), a WHO “Watch” status antibiotic, with lower respiratory infection (LRI) (*n* = 761, 33%), bloodstream infection (*n* = 426, 18%), and prophylaxis (*n* = 402,17%), as the most frequently identified indications for treatment (Table 3). For all indications for treatment, Ceftriaxone was the most frequently prescribed antibiotic, either alone or in combination with metronidazole, except for in upper respiratory infections, where amoxicillin-clavulanate was more frequently used. Metronidazole, also a WHO “Watch” status antibiotic, was the second-most widely used in total, but most often in combination with ceftriaxone for all indications, particularly for gastrointestinal (GI) infections (*n* = 87/218, 40%), bloodstream infections (*n* = 79/251, 31%), combined lower respiratory plus blood stream infection (*n* = 32/64, 50%), and combined neurologic infection plus LRI, GI or bloodstream infection (*n* = 36/70, 51%) (Tables 2 and 3).

**Table 2:** Patient demographics and prescription practices for children receiving antibiotics in the emergency medicine department

Characteristic	*N* = 2324
Age in years	n (%)
<2	1037 (45)
≥2 to < 5	690 (30)
5 to < 12	431 (18)
12 to 14	158 (7)
Missing/unknown	8 (0)
Sex	
Male	1349 (58)
Number of antibiotics received	
1	1612 (69)
2	668 (39)
3	14 (1)
4	1 (0)
Missing/unknown	29 (1)
Antibiotic given	
Ceftriaxone (w)	1653 (72)
Metronidazole (w)	660 (29)
Amoxicillin-Clavulanate (a)	444 (19)
Azithromycin (w)	53 (2)
Ampicillin-cloxacillin (nr)	45 (2)
Ciprofloxacin (w)	36 (2)
Other*	42 (2)
Indication for antibiotic use	
Lower respiratory infection	761 (33)
Bloodstream infection	426 (18)
Prophylaxis	402 (17)
Upper respiratory infection	376 (16)
Gastrointestinal infection	351 (15)
Other^†^	487 (21)
Patient disposition	
Admitted to other ward^α^	863 (37)
Discharged	710 (31)
Admitted to pediatric ward	676 (29)
Died	30 (1)
Transferred	22 (1)
Other^‡^	15 (1)

a = WHO AWaRE Access status; w = WHO AWaRE Watch status; nr = not recommended for use by WHO AWaRE

*Other included cefixime, amoxicillin, penicillin V, gentamicin, ampicillin, doxycycline, cefotaxime, cefepime, and clindamycin

†Other included neurological infection, urinary tract infection, skin infection, unknown source of infection, musculoskeletal infection, malaria, ophthalmologic infection, tuberculosis, and oncology-related illness

^α^Including but not limited to pediatric critical care, malnutrition ward, gastroenteritis ward, surgical wards

‡Other included left against medical advice and unknown

**Table 3:** The number and proportion of the most frequently used antibiotics in the Muhimbili National Hospital Emergency Medicine Department for pediatric patients, by the most common indication or combination of indications for use. Indication-for-use categories are mutually exclusive, with n representing the number of patient encounters

Indication for use	Antibiotic	*n* (%)
Lower respiratory infection (LRI) (*n* = 532)	Ceftriaxone	386 (73)
	Ceftriaxone + metronidazole	70 (13)
	Amoxicillin-clavulanate	49 (9)
	Other^†^	27 (5)
LRI + bloodstream infection (*n* = 64)	Ceftriaxone + metronidazole	32 (50)
	Ceftriaxone	25 (39)
	Other	6 (9)
LRI + GI infection (*n* = 42)	Ceftriaxone	21 (50)
	Ceftriaxone + metronidazole	14 (33)
	Other	7 (17)
Prophylaxis^#^ (*n* = 380)	Ceftriaxone	148 (39)
	Ceftriaxone + metronidazole	134 (35)
	Amoxicillin-clavulanate	37 (10)
	Other	49 (13)
Upper respiratory infection (*n* = 277)	Amoxicillin-clavulanate	169 (61)
	Ceftriaxone	33 (12)
	Azithromycin	23 (8)
	Other	52 (19)
Bloodstream infection (*n* = 251)	Ceftriaxone	129 (51)
	Ceftriaxone + metronidazole	79 (31)
	Amoxicillin-clavulanate	22 (9)
	Other	21 (8)
Gastrointestinal (GI) infection (*n* = 218)	Ceftriaxone + metronidazole	87 (40)
	Ceftriaxone	60 (28)
	Metronidazole	29 (13)
	Amoxicillin-clavulanate	13 (6)
	Other	29 (13)
Neurologic infection (*n* = 85)	Ceftriaxone	54 (64)
	Ceftriaxone + metronidazole	29 (34)
	Other	2 (2)
Neurologic + LRI/GI/Bloodstream^θ^ (*n* = 70)	Ceftriaxone + metronidazole	36 (51)
	Ceftriaxone	33 (47)
	Other	2 (3)
Urinary infection (*n* = 72)	Ceftriaxone	30 (42)
	Amoxicillin-clavulanate	20 (28)
	Other	16 (22)
All other indications^¥^ (*n* = 333)	Ceftriaxone	124 (37)
	Ceftriaxone + metronidazole	70 (24)
	Amoxicillin-clavulanate	63 (19)
	Other	76 (23)
		Total *N* = 2324

^†^ Other antibiotics used include (for all indications): ampicillin-cloxacillin, amoxicillin, azithromycin, cefixime, cefotaxime, ciprofloxacin, doxycycline, gentamicin, metronidazole, Penicillin V, unlisted, and combinations of these

^#^Prophylaxis indications included trauma, surgery, high-risk conditions (e.g., suspected or known oncologic process or immunosuppression, complex medical history)

^θ^Includes neurologic indication plus any combination of LRI, GI, or bloodstream infection

^¥^Other indications included musculoskeletal, skin and soft tissue, other, and unknown source of infection, or any combination of the above-listed indications not already included in the table

### Culture isolates

A total of 4783 microbiologic samples from 3312 unique patients were processed in the MNH lab between March 2022 and February 2023. These included 1195 urine samples, 2971 blood samples, and 617 other samples (e.g., wound cultures) from all pediatric patients. After removal of duplicate samples and those not from blood or urine, a total of 3,370 samples were included in the analysis.

A total of 292 pathogens were isolated from 3,380 samples (8.6% positivity), 100 from urine and 192 from blood. Of these, 265 were from unique individuals, 90 from urine and 175 from blood, of which *Klebsiella pneumoniae* (*n* = 59, 22%), *Escherichia coli* (*n* = 53, 20%), and *Staphylococcus aureus* (*n* = 39, 15%) were the most frequently identified organisms. *K. pneumoniae* was the most common organism isolated from blood samples (*n* = 42, 24%), while *E. coli* was the most frequent from urine samples (*n* = 35, 39%). Most samples were sent from patients admitted to inpatient units (*n* = 1839, 56%), with the majority from the General Pediatric Ward (*n* = 1271, 38%), followed by the Intensive Care Unit (ICU) (*n* = 537, 16%). The General Pediatric Ward was also the most common location for patients with a positive culture sample (*n* = 121, 46%) (Table 4). Coagulase-negative Staphylococcus species were the most frequently isolated contaminants.

**Table 4:** Summary of characteristics for pediatric blood and urine culture samples at MNH from 2022–2023. Note that N represents individual patients, not samples, with only the first sample included per patient

			Positive Samples		
	All patients	Negative*	Blood	Urine	Overall
Characteristics	*N* = 3312	*N* = 3047	*N* = 175	*N* = 90	*N* = 265
Age (years),					
median (IQR)	1.0 (0.1, 6.0)	1.0 (0.0, 6.0)	1.0 (0.6, 6.0)	4.0 (1.0, 7.0)	2.0 (0.8, 7.0)
Sex, n(%)					
Female	1791 (54)	1653 (54)	96 (55)	42 (47)	138 (52)
Male	1517 (46)	1391 (46)	78 (45)	48 (53)	126 (48)
Unknown	4	3	1	n/a	1
Location type, n(%)					
Inpatient ward	1,839 (56)	1709 (56)	82 (47)	48 (53)	130 (49)
Outpatient	896 (27)	808 (27)	48 (27)	40 (44)	88 (33)
Intensive Care	570 (17)	570 (17)	45 (26)	2 (2.2)	47 (18)
Specific department, n(%)					
General Pediatric	1271 (38)	1150 (38)	69 (39)	52 (58)	121 (46)
Outpatient Dept	470 (14)	413 (14)	39 (22)	18 (20)	57 (22)
Intensiive care	537 (16)	499 (16)	36 (21)	2 (2.2)	38 (14)
Burn Ward	39 (1)	8 (0)	22 (13)	9 (10)	31 (12)
Medical Ward	100 (3)	92 (3)	4 (2)	4 (4)	8 (3)
EMD	69 (2)	63 (2)	4 (2)	2 (2)	6 (2)
Other	826 (25)	822 (27)	1 (1)	3 (3)	4 (3)
Gram Stain, n(%)					
Gram -	--	--	136 (78)	88 (98)	224 (85)
Gram +	--	--	39 (22)	2 (2)	41 (15)
Organism isolated, n(%)					
*K. pneumoniae*	--	--	42 (24)	17 (19)	59 (22)
*E. coli*	--	--	18 (10)	35 (39)	53 (20)
*S.aureus*	--	--	38 (22)	1 (1)	39 (15)
*P. aeruginosa*	--	--	19 (11)	8 (8.9)	27 (10)
*A. baumannii*	--	--	12 (7)	2 (2)	14 (5)
*Acinetobacter sp.*	--	--	12 (7)	1 (1)	13 (5)
*K. oxytoca*	--	--	2 (1)	9 (10)	11 (4)
*S. marcescens*	--	--	4 (2)	3 (3)	7 (3)
Other			28 (16)	14 (16)	42 (16)

Other organisms include: Citrobacter, Enterobacter, Serratia, Enterococcus, Pseudomonas, Proteus, Pantoea, Kluyvera, and Klebsiella species

*EMD *Emergency Medical Department

*Negative designation also includes all samples classified as contaminants

### Susceptibility profiling

Gram-negative bacteria demonstrated low susceptibility to third- and fourth-generation cephalosporins. Only 18% (*n* = 9/51) of *E. coli* isolates and 20% (*n* = 10/49) *K. pneumoniae* isolates were susceptible to ceftriaxone, with 19% (*n* = 11/58) of *E. coli* and 21% (*n* = 13/64) of *K. pneumoniae* isolates susceptible to ceftazidime and 17% (*n* = 10/59) of *E.coli* and 21% (*n* = 12/61) of *K. pneumoniae* isolates with susceptibility to cefepime. Similar susceptibilities were present for sulfonamides (21% [*n* = 11/53] for *E. coli* and 26% [*n* = 15/57] for *K. pneumoniae* to trimethoprim-sulfamethoxazole), beta-lactams/beta-lactamase inhibitors (20% [*n* = 10/49] for *E. coli*, 18% [*n* = 10/55] for *K pneumoniae* to piperacillin-tazobactam), and fluoroquinolones (20% [*n* = 11/54] for *E. coli* and 22% [14/63] for *K. pneumoniae* to ciprofloxacin). Resistance to aminoglycosides was slightly lower, with 40% (*n* = 23/57) of *E. coli* and 25% (*n* = 15/60) of *K. pneumoniae* susceptible to gentamicin, and 49% (*n* = 29/59) of *E. coli* and 49% (*n* = 31/63) of *K. pneumoniae* susceptible to amikacin. There was a higher overall susceptibility to carbapenems, with 81% (*n* = 47/58) and 71% (*n* = 44/62) of E. coli and K. pneumoniae, respectively, being susceptible to meropenem (Table 5). *E. coli* from urine samples had 88% (*n* = 30/34) susceptibility to nitrofurantoin. Other Enterobacterales and *K. pneumoniae* from urine had sensitivity of 80% (*n* = 16/20) and 42% (*n* = 8/19), respectively; however, the total number of isolates tested for both groups was under 30.

**Table 5:** Antibiogram (percent susceptibility) of the most commonly isolated bacteria from blood and urine samples from pediatric patients at MNH to antibiotic agents. Not all isolates were tested against all agents due to intermittent reagent stock-outs. The number susceptible over the total tested is provided in parentheses. Note that N is at the isolate, not patient, level

Antibiotic	% Susceptible (*n*/total tested)*					
	S. aureus	E. coli	K. pneumoniae	Other Enterobacterales^†^	Acinetobacter spp.	*P*. aeruginosa
	*N* = 39	*N* = 59	*N* = 64	*N* = 57	*N* = 31	*N* = 29
Penicillin	5 (1/19)	--	--	--	--	--
Ampicillin	--	4 (2/57)	3 (2/60)	--	--	--
Oxacillin^α^	**41 (15/37)**	--	--	--	--	--
Erythromycin	30 (11/37)	--	--	--	--	--
Clindamycin	76 (29/38)	--	--	--	--	--
TMP-SMX	56 (20/36)	21 (11/53)	26 (15/57)	42 (22/52)	44 (12/27)	--
Ceftriaxone	--	**18 (9/51)**	**20 (10/49)**	38 (16/42)	31 (5/16)	--
Ceftazidime	--	19 (11/58)	20 (12/61)	41 (22/54)	38 (11/29)	74 (20/27)
Pip-tazo	--	20 (10/49)	18 (10/55)	25 (13/52)	48 (13/27)	52 (12/23)
Meropenem	--	**81 (47/58)**	**71 (44/62)**	69 (36/52)	61 (19/31)	78 (21/27)
Cefepime	--	18 (10/57)	21 (13/61)	42 (21/50)	55 (11/20)	88 (23/26)
Gentamicin	35 (13/37)	40 (23/57)	25 (15/60)	50 (27/54)	52 (14/27)	--
Ciprofloxacin	44 (17/39)	20 (11/54)	22 (14/63)	35 (19/54)	38 (11/29)	48 (14/29)
Amikacin	--	49 (29/59)	49 (31/63)	57 (32/56)	54 (13/24)	79 (23/29)
Nitrofurantoin^‡^	--	88 (30/34)	42 (8/19)	80 (16/20)	--	--

Bold text indicates guideline-recommended or commonly used agents, commonly isolated, or more clinically relevant pathogens.

*TMP-SMX *Trimethoprim-sulfamethoxazole, *Pip-tazo *piperacillin-tazobactam.

*N is at the isolate level and includes results from 14 patients with > 1 positive isolate; only first positive isolate per patient per species used

-- indicates agent not tested/not applicable to isolate.

^**α**^Cefoxitin used as a surrogate for methicillin/oxacillin resistance per CLSI guidelines.

^†^Other Enterobacterales include: *Citrobacter spp.*,* Enterobacter spp.*,* other Klebsiella spp.*,* Pantoea spp*,* Proteus spp.*,* Serratia spp.*, and *Kluyvera spp. (*does not include *Salmonella).*

^‡^ Tested only isolates from urine.

Acinetobacter isolates also had reduced susceptibility across antibiotic agents of multiple different classes including ceftriaxone (31%, *n* = 5/16), ciprofloxacin (38%, *n* = 11/29), trimethoprim-sulfamethoxazole (44%, *n* = 12/27), piperacillin-tazobactam (48%, *n* = 13/27), and aminoglycosides (gentamicin: 52%, 14/27; amikacin: 54%, *n* = 13/24). *P. aeruginosa* demonstrated high frequencies of susceptibility to most anti-pseudomonal antibiotic agents tested, including cefepime (88%, *n* = 23/26), amikacin (79%, *n* = 23/29), meropenem (78%, *n* = 21/27), and ceftazidime (74%, *n* = 20/27). Of note, for both *Acinetobacter* and *Pseudomonas* isolates, counts for all agents included in our antibiogram were below the threshold of 30. For gram-positive organisms, *S. aureus* had limited susceptibility to oxacillin (41%, *n* = 15/37), but most isolates were susceptible to clindamycin (76%, *n* = 29/38) (Table 5).

The majority of Enterobacterales isolated met criteria for classification as MDR organisms (*n* = 142, 93%). A smaller fraction of *P. aeurginosa* (*n* = 5, 19%) and *S. aureus* isolates (*n* = 25, 64%) also met these criteria. Multi-drug resistance was observed across all patient ages, sexes, and patient locations at the time of sample procurement (Table 6).

**Table 6:** Multi-drug resistance (MDR) classification for Enterobacterales, P. aeruginosa, and S. aureus isolates using European Centre for Disease Prevention and Control and Centers for Disease Control and Prevention criteria, by patient age, gender, and hospital location at the time culture samples were collected. Only data from the first positive isolate by date was included for participants with multiple positive samples

	Enterobacterales^†^, *N* = 153		*P*. aeruginosa, *N* = 27		S. aureus, *N* = 39	
	MDR	non-MDR	MDR	non-MDR	MDR	non-MDR
Characteristic	*N* = 142, 93%	*N* = 11, 7%	*N* = 5, 19%	*N* = 22, 81%	*N* = 25, 64%	*N* = 14, 36%
Age, median (IQR)	2.0 (0.8, 6.0)	3.0 (0.6, 6.0)	3.0 (0.5, 7.0)	3.5 (1.0, 6.0)	2.0 (1.0, 4.0)	5.5 (2.0, 13.0)
Sex, n(%)						
Male	69 (49)	5 (45)	5 (100)	9 (41)	13 (52)	10 (71)
Female	72 (51)	6 (55)	0	13 (59)	12 (48)	4 (29)
Unknown	1	0	n/a	n/a	n/a	n/a
Patient Location, n(%)						
Inpatient	72 (51)	4 (36)	1 (20)	14 (64)	14 (56)	8 (57)
ICU	24 (17)	1 (9)	2 (40)	2 (9)	1 (4)	3 (21)
Outpatient	46 (32)	6 (55)	2 (40)	6 (27)	10 (40)	3 (21)

*MDR *Multi-drug resistant, *MRSA *methicillin-resistant S. aureus, *MSSA *methicillin-sensitive *S.aureus, ICU *intensive care unit, *IQR *interquartile range

^†^Excludes salmonella and pseudomonal species

## Discussion

We characterized current guidelines for treatment of common infections, empiric antibiotic prescriptions, microbiology results, and resistance profiles for pediatric patients at a tertiary care hospital in Tanzania. We found that ceftriaxone, considered a “Watch” status antibiotic by the WHO, was used in over 70% pediatric patients who received empiric antibiotics in the MNH EMD, particularly for the treatment of lower respiratory infections. We also demonstrated a high prevalence of AMR in gram-negative organisms, including only 18% susceptibility to ceftriaxone among 51 total *E. coli* isolates from blood and urine in this population of Tanzanian children. In addition, a minority fraction of both *E. coli* and *K. pneumoniae* isolates were susceptible to gentamicin (40% and 25%, respectively). While susceptibility to meropenem, a WHO “Reserve” status antibiotic, was much higher (81% of 59 *E. coli* isolates and 71% of 64 *K. pneumoniae* isolates), this is still concerning given its infrequent use clinically as a second-line agent, likely due to comparatively higher cost and reduced availability in this setting. We also found evidence of methicillin resistance in *S. aureus* (41% of 37 samples).

Commonly reported antibiotic agents for the treatment of pediatric infections used by providers in SSA vary across the existing literature, depending on availability and local epidemiology; however, the general trend is that antibiotic use (and, similarly, AMR) is increasing [1]. Similar to our findings, prior studies in Tanzania and elsewhere in SSA have reported a high frequency of ceftriaxone for the empiric management of pediatric infections, primarily among hospitalized patients [7, 30, 32, 33]. Ceftriaxone’s widespread availability, relatively low cost, use as an intravenous or intramuscular agent, once-daily dosing, and coverage of a variety of bacterial pathogens associated with infectious syndromes in pediatric patients make it an attractive choice for empiric coverage [34]. Qualitative studies in similar settings have revealed that clinicians often use empiric antibiotic treatment when the source of fever cannot be easily identified, due to concerns about inadequate availability of confirmatory testing and patient follow-up, fear of caregiver dissatisfaction, or the potential for undertreatment and subsequent poor outcomes [13, 14, 35]. 

In our study, treatment patterns seemed to be dictated by local empiric pediatric sepsis guidelines in the EMD rather than by the national Tanzanian STG’s disease-specific empiric antibiotic treatment recommendations for sepsis. The local EMD sepsis guidelines recommend universal treatment with ceftriaxone and metronidazole for all children with at ≥ 2 criteria for systemic inflammatory response syndrome (SIRS) for age (i.e. elevated heart rate, respiratory rate, temperature, low blood pressure, or elevated white blood cell count), regardless of likely source of infection (Table 1), vs. the Tanzanian STG, which use the Quick Sequential Organ Failure Assessment (qSOFA) score for adults (i.e., altered mental status, hypotension, elevated respiratory rate) and do not have pediatric-specific sepsis criteria [25]. For both, while recommendations for second-line or alternative treatment are provided, there is no specific guidance for when to escalate or switch to a second-line treatment option based on clinical risk factors present on initial presentation. When guidance is present, it relies on the receipt of culture results or a lack of improvement after a longer time period (e.g., 24 h). These challenges are similar to those identified elsewhere in SSA, including conflicting definitions of pediatric sepsis and limited consensus antibiotic guidelines, particularly in higher-level healthcare facilities where susceptibility to first-line agents may be lower [36, 37]. This emphasizes a need for accessible, locally specific antimicrobial data to guide treatment recommendations by facility level.

As trainees primarily staff the EMD, providers on the MNH AMS committee have speculated that resident providers may prefer the EMD sepsis guidelines and their broader inclusion criteria due to fear of repercussions or negative feedback by their supervisors should a patient’s condition deteriorate. This leaves room for quality initiatives incorporating provider training on the importance of narrow-spectrum antibiotic use and revision of local guidelines to provide clear recommendations on risk factors for severe infection, clinical criteria for treatment failure in referred or pretreated patients, and differentiation of when to escalate to second-line options, while reflecting upon and incorporating local epidemiologic and microbiologic profiles.

The overall frequency of positive blood and urine cultures at the MNH lab was 8.6%, comparable to those found in similar cohorts of children [38–42]. Our susceptibility profile results included both gram-positive and gram-negative organisms resistant to several classes of antibiotics, including third- and fourth-generation cephalosporins, aminoglycosides, fluoroquinolones, and beta-lactams/beta-lactamase inhibitors (Table 5). There was also evidence of developing resistance to broader-spectrum agents, including over 25% of K. pneumoniae isolates resistant to meropenem. As microbiologic capacity is often concentrated on admitted patients in referral hospitals, our resistance patterns are similar to other studies in tertiary care facilities in Africa; however, these other study populations predominantly consisted of sicker cohorts of participants meeting sepsis criteria or needing ICU-level care, with higher overall rates of culture positivity than in our cohort [39, 42–44]. 

Though the general microbiology lab does not have capacity for more extensive resistance profiling, previous data from higher-capacity academic laboratories of pediatric populations in SSA, particularly in Tanzania, have also revealed elevated levels of ceftriaxone resistance [3, 38–40] and ESBL-producing gram-negative organisms [3, 45–47]. Others have found MRSA is common even in pediatric patients [15–17, 19, 20]. We found evidence of multidrug resistance in samples drawn from both the inpatient and outpatient departments (Table 6). Still, we lacked sufficient contextual data to conclude whether these represented primarily community- or nosocomial-transmitted pathogens. Given that MNH is a tertiary hospital and referral center, this population may have a higher likelihood of prior antibiotic exposure, colonization, and nosocomial infection than the general population. Thus, additional studies are needed to provide a detailed review of medical trajectory (including referral course), hospital length of stay, prior culture results, antibiotic exposure, and preexisting medical conditions, to balance judicious antibiotic use recommendations with access to reserve-level antibiotics for high-risk patients who truly need them.

These trends in antibiotic susceptibility are concerning, given that ceftriaxone was the most commonly empiric treatment regardless of presumed source of infection. This is consistent with prior studies in Tanzania and likely a contributor to the resistance patterns observed in our study [48–52]. While meropenem and other more broad-spectrum agents (e.g., colistin) are available as second-line agents, these typically require pharmacy approval for use based on culture results, which are not available to clinicians working in the empiric/emergency setting. Further context is needed to understand which clinical risk factors may be associated with AMR, the rates of undertreatment versus treatment failure with first-line agents, and when broadening empiric coverage should be considered before culture results are available.

Our study has several limitations. First, this was a single-center study, which limits generalizability. Second, this study did not correlate antibiotic prescriptions, microbiology lab results, clinical course, or outcomes at the patient level; thus, we were unable to determine the potential effects of pretreatment, suitability of empiric antibiotics used, or other necessary factors to comprehensively characterize contributors to AMR in this setting. Third, despite recent funding for capacity building in the microbiology lab, regents and supplies are still limited, thus not all isolates were tested against recommended agents, including ceftriaxone, and our antibiogram did not include further typing of ESBL-producing organisms, confirmation of methicillin-resistance for *S. aureus*, or characterization of other resistance mechanisms due to lack of capacity.

Our findings highlight the high incidence of ceftriaxone as an empirical antibiotic choice and the high frequency of resistance to commonly available antibiotics among gram-negative organisms in a pediatric tertiary care hospital in Tanzania. In practice, provider antibiotic prescribing patterns were consistent with broad local sepsis guidelines based on SIRS criteria rather than the narrower, disease-specific national treatment guidelines. Our findings call for quality improvement initiatives to support AMS programs in providing ongoing review of antibiotic use and microbiologic lab data, increasing provider education and incorporating microbiologic results into clinical management decisions, and creating clinical guidelines responsive to local epidemiology and specific patient risk factors. Actionable next steps include reviewing available medical records data for children with positive culture results, evaluating utilization patterns for microbiologic testing and results by providers, and collecting provider and trainee feedback on current AMS programming for future curriculum development. Dedicated government funding is needed to support pediatric AMS programs and region-specific surveillance of antibiotic use and bacterial resistance patterns.

## Supplementary Information

Supplementary Material 1: Supplementary Table 1. MNH Central Processing Lab Antibiotic Susceptibility Testing Workshe.

## Data Availability

The data that support the findings of this study are available from Muhimbili National Hospital. Still, these data are subject to restrictions on availability and were used with the hospital’s permission for the current study and are therefore not publicly available. Data are, however, available from the authors upon reasonable request and with permission of Muhimbili National Hospital.

## Abbreviations

AMR: Antimicrobial resistance
AMS: Antimicrobial stewardship
BD: Beckton Dickinson
EMD: Emergency Medicine Department
EMR: Electronic medical record
ESBL: Extended-spectrum beta-lactamase
IRB: Institutional Review Board
LMICs: Low- and middle-income countries
MNH: Muhimbili National Referral Hospital
MRSA: Methicillin-resistant Staphylococcus aureus
MUHAS: Muhimbili University of Health and Allied Sciences
SSA: Sub-Saharan Africa
WHO: World Health Organization
