# Supplementary material for: Pediatric empiric antibiotic use and resistance patterns at a single tertiary care hospital in Tanzania
Source: BMC Pediatr. 2026 Jul 8;26:956. doi: 10.1186/s12887-026-07261-z (PMC13640168; doi:10.1186/s12887-026-07261-z)
Supplement: Supplementary file 1 [file 12887_2026_7261_MOESM1_ESM.pdf]

Supplemental File 1: Muhimbili Central Pathology Lab Antibiotic Susceptibility Testing Worksheet

Muhimbili National Hospital-Central Pathology Laboratory, Kalenga Street, Ilala District, P.O.BOX 65002, Dar Es Salaam, Phone No +255222151367-9, Fax+255222150534		
Document name	Microbiology culture work sheet	
Document number	CPL BACT TF 008	
Version number	Version 05	
Control copy number	Not applicable	
Effective date	01/05/2024	
Review date	30/04/2027	
Section	Bacteriology	

Performed by		

FINAL CULTURE REPORT:

SUSCEPTIBILITY TEST RESULTS:

Antibiotic (µg)	Zone size (mm)	Interp. (S, I, R)	Antibiotic (µg)	Zone size (mm)	Interp. (S, I, R)
**A. GRAM NEGATIVE (Enterobacteriacae)**			**C. GRAM POSITIVE (Staphylococcus)**		
Gentamycin 10µg			Cefoxitin 30µg		
Amikacin 30µg			Clindamycin 2µg		
Chloramphenical 30µg			Gentamycin 10µg		
Ceftriaxone			Doxycycline 30µg /Tetracycline30µg		
Amoxy Clav. 20/10µg			Ciprofloxacin 5µg		
Ceftazidime 30µg			Trimeth/Sulfa (SXT) 1.25/23.75µg		
Cefepime 30µg			Penicillin G 10units		
Aztreonem 30µg			Azithromycin/Erythromycin		
Cefoxitin 30µg			Chloramphenical 30µg		
Aztreonem 30µg			**MRSA: Pos.** ☐ **Neg.** ☐	N/A	N/A
Tobramycin 10µg			**ICR: Pos.** ☐ **Neg.** ☐	N/A	N/A
Doxycycline 30µg/Tetracycline30µg			**D. OTHER GRAM POSITIVE(Streptococcus/Enterococcus)**		
Trimeth/Sulfa (SXT)1.25/23.75µg			Penicillin G 10units		
Piperacillin /Tazobactam 100/10µg			Vancomycin 30 µg		
Nitrofurantoin 300µg (Urine only)			Clindamycin 2µg		
Ciprofloxacin 5µg			Ampicillin 10µg		
Nalidixic Acid 30µg (Urine only)			Oxacillin		
Meropenem/Imipenem 10µg			Erythromycin 15µg		
**B. PSEUDOMONAS SPP.**			Tetracycline 30µg (in Urine only)		
Tobramycin 10µg			Doxycycline 30µg (DOX)		
Ciprofloxacin 5µg			Ciprofloxacin 5µg (in Urine only)		
Imipenem/Meropenem 10µg			**E. HAEMOPHILUS SPP.**		
Amikacin 30µg (AK Pseudo in urine only)			Penicillin G 10units		
Ceftazidime 30µg			Chloramphenical 30µg		
Cefepime 30µg (FEP)			Trimeth/sulfa(SXT)1.25/23.75µg		
Piperacillin /Tazobactam 100/10µg			Clarithromycin 15 µg		
Aztreonem 30µg			Rifampin 5 µg (RIF)		
Piperacillin 100µg			Tetracycline 30µg		
**ESBL: Pos.** ☐ **Neg.** ☐	NA	NA	**ID Factor v**		
**CRE: Pos.** ☐ **Neg.** ☐	NA	NA	**ID Factor v+x**		

Note: This is uncontrolled copy
